# Proteomic and transcriptomic profiling reveals a link between the PI3K pathway and lower estrogen-receptor (ER) levels and activity in ER+ breast cancer

**DOI:** 10.1186/bcr2594

**Published:** 2010-06-22

**Authors:** Chad J Creighton, Xiaoyong Fu, Bryan T Hennessy, Angelo J Casa, Yiqun Zhang, Ana Maria Gonzalez-Angulo, Ana Lluch, Joe W Gray, Powell H Brown, Susan G Hilsenbeck, C Kent Osborne, Gordon B Mills, Adrian V Lee, Rachel Schiff

**Affiliations:** 1 Dan L. Duncan Cancer Center, Baylor College of Medicine, One Baylor Plaza, BCM 600, Houston, TX 77030, USA; 2 Lester and Sue Smith Breast Center, Baylor College of Medicine, One Baylor Plaza, BCM 600, Houston, TX 77030, USA; 3 Department of Systems Biology, M.D. Anderson, Houston, TX 77030, USA; 4 Department of Medicine, Baylor College of Medicine, One Baylor Plaza, BCM 600, Houston, TX 77030, USA; 5 Department of Molecular and Cellular Biology, Baylor College of Medicine, One Baylor Plaza, BCM 600, Houston, TX 77030, USA; 6 Department of Breast Medical Oncology, M.D. Anderson, Houston, TX 77030, USA; 7 Department of Hematology Oncology, Hospital Clinico Universitario de Valencia, Av de Vicente Blasco Ibáñez, 17, 46010, Valencia, Spain; 8 Life Sciences Division, Lawrence Berkeley National Laboratory, One Cyclotron Road, Berkeley, CA 94720, USA; 9 Department of Laboratory Medicine, UCSF Helen Diller Family Comprehensive Cancer Center, University of California, 1600 Divisadero Street, San Francisco, CA 94143, USA; 10 Department of Clinical Cancer Prevention, Division of OVP, Cancer Prevention and Population Sciences, M.D. Anderson, Houston, TX 77030, USA

## Abstract

**Introduction:**

Accumulating evidence suggests that both levels and activity of the estrogen receptor (ER) and the progesterone receptor (PR) are dramatically influenced by growth-factor receptor (GFR) signaling pathways, and that this crosstalk is a major determinant of both breast cancer progression and response to therapy. The phosphatidylinositol 3-kinase (PI3K) pathway, a key mediator of GFR signaling, is one of the most altered pathways in breast cancer. We thus examined whether deregulated PI3K signaling in luminal ER^+ ^breast tumors is associated with ER level and activity and intrinsic molecular subtype.

**Methods:**

We defined two independent molecular signatures of the PI3K pathway: a proteomic (reverse-phase proteomic array) PI3K signature, based on protein measurement for PI3K signaling intermediates, and a PI3K transcriptional (mRNA) signature based on the set of genes either induced or repressed by PI3K inhibitors. By using these signatures, we scored each ER^+ ^breast tumor represented in multiple independent expression-profiling datasets (four mRNA, n = 915; one protein, n = 429) for activation of the PI3K pathway. Effects of PI3K inhibitor BEZ-235 on ER expression and activity levels and cell growth were tested by quantitative real-time PCR and cell proliferation assays.

**Results:**

Within ER^+ ^tumors, ER levels were negatively correlated with the PI3K activation scores, both at the proteomic and transcriptional levels, in all datasets examined. PI3K signature scores were also higher in ER^+ ^tumors and cell lines of the more aggressive luminal B molecular subtype versus those of the less aggressive luminal A subtype. Notably, BEZ-235 treatment in four different ER^+ ^cell lines increased expression of *ER *and ER target genes including *PR*, and treatment with IGF-I (which signals via PI3K) decreased expression of *ER *and target genes, thus further establishing an inverse functional relation between ER and PI3K. BEZ-235 had an additional effect on tamoxifen in inhibiting the growth of a number of ER^+ ^cell lines.

**Conclusions:**

Our data suggest that luminal B tumors have hyperactive GFR/PI3K signaling associated with lower ER levels, which has been correlated with resistance to endocrine therapy. Targeting PI3K in these tumors might reverse loss of ER expression and signaling and restore hormonal sensitivity.

## Introduction

Hormone therapy for breast cancer represents one of the earliest targeted therapies and continues to be one of the most effective therapies in breast cancer [1]. However, only about 60% to 70% of patients with ER^+ ^tumors respond to therapy [2]. Given that the majority of diagnosed breast cancers are ER^+^, this leaves a large subset of breast cancers that do not respond to hormone therapy and are subsequently often treated with chemotherapy. Basic and clinical studies have shown the critical importance of the steroid receptor estrogen receptor (ER) and progesterone receptor (PR) in the development of the normal mammary gland and in the development and progression of breast cancer [3,4]. Loss or reduced expression of either of these receptors is associated with worse prognosis and reduced response to antiestrogen therapy [5]. It also has become clear that both levels and activity of ER and PR are dramatically influenced by growth factor receptor (GFR) signaling pathways and that this crosstalk is a major determinant of both breast cancer progression and response to therapy [6,7].

Early studies identified PI3K activity associated with viral oncogenes and led to its identification as a major signaling pathway in cancer and a key mediator of GFR signaling [8-10]. The PI3K pathway is now recognized to be one of the most altered pathways in human breast cancer. For example, breast tumors show mutation or loss of PTEN or both, amplification and activating mutations in PIK3CA, amplification of Akt2 and p70S6kinase, and overexpression of Akt3 [11]. When the more-global picture of upstream and downstream PI3K signaling is taken into account (for example, amplification or mutation of upstream GFRs, mutation of insulin receptor substrates (IRSs), and mutation of NF-κB), this points to the PI3K pathway as being one of the most critical determinants in breast cancer initiation and progression [12]. Consistent with the mutational spectrum of PI3K signaling intermediates in breast cancer, direct analysis of PI3K activation has shown an association with poor outcome [13-19]. Similarly, loss of PTEN is associated with low ER and PR and poor outcome [20-22]. A recent report showed the significance of downregulation of key molecules in the PI3K pathway in response to aromatase-inhibitor therapy, further emphasizing the predictive and therapeutic role of this pathway in hormonal therapy [23].

In this study, we addressed the question whether elevated PI3K decreases ER levels and activity to cause hormone resistance within the ER^+ ^subset of human breast cancer. We hypothesized that this loss of ER expression or function or both may be reversed by inhibition of PI3K, which might allow better subsequent therapeutic targeting by using a combination of PI3K inhibitors and antiestrogens. Our approach in examining human breast tumors and cell lines was to use gene-expression and proteomic profiling data to define molecular signatures of PI3K and then to use these signatures as a surrogate for PI3K activity. PI3K signaling is manifested at both protein and transcription levels, whereby the signal initiated by GFR is transduced by phosphorylation of signaling proteins, eventually leading to changes in gene transcription. Therefore, we defined two different PI3K molecular signatures: (a) a PI3K protein signature (by reverse-phase protein arrays, or RPPAs), and (b) a PI3K mRNA signature (by gene-expression array). Interestingly, both of these signatures yielded similar associations in the human tumor datasets examined.

## Materials and methods

### Human breast tumor samples

The human ER^+ ^breast tumors were obtained from tumor banks after pathologist review under the auspices of Institutional Review Board-approved protocols at Hospital Clinico Universitario de Valencia (Valencia, Spain), the University of Texas M. D. Anderson Cancer Center, and Baylor College of Medicine. Informed consent was obtained from all patients involved. Preparation of the tumor samples for protein analysis and characterization of ER status was carried out as previously described [24].

### Reverse phase proteomic arrays

RPPA, as performed in our group, has been described previously [25,26] and was used to quantify PTEN expression and phosphorylation of AKT at Thr^308 ^and Ser^473^, glycogen synthase kinase 3 (GSK3) at Ser^21^, mammalian target of rapamycin (mTOR) at Ser^2448^, and p70S6K at Thr^389 ^as a ratio to total expression of each protein by using antibodies from cell signaling (total PTEN and all phosphospecific antibodies). For each protein, normalized expression values were centered across the ER^+ ^tumors on the mean (overall findings observed were the same when centering values on the median versus the mean; results not shown). The protein lysates from breast cancer cell lines were obtained from the Lawrence Berkeley National Laboratory at the University of California at San Francisco.

### Gene-expression analysis

Gene-transcription profiling datasets were obtained from previous studies (CMap build01, [GEO:GSE5258]; van de Vijver (available at [27]); Loi, [GEO:GSE9195]; Wang, [GEO:GSE2034]; Desmedt, [GEO:GSE7390]; Neve (available at [28]). Of the 134 ER^+ ^tumors in the Desmedt dataset, 28 were also represented in the Loi dataset, and so these were removed before computing the correlations for Desmedt. The CMap dataset values were processed as previously described [29]. Differentially expressed genes were identified by using a two-sided *t *test on log-transformed data, with the false discovery rate (FDR) estimated by using the method of Storey *et al*. [30]. Java TreeView [31] represented expression values as color maps. To score each ER^+ ^breast tumor for similarity to our PI3K transcription signature, we derived a ''*t *score'' for the tumor in relation to the PI3K signature patterns, as previously described [32,33]. In brief, the PI3K mRNA *t *score was defined as the two-sided *t *statistic comparing the average of the PI3K-induced genes with that of the repressed genes within each tumor (after first centering the log-transformed values on the median across samples; Loi dataset: values centered to standard deviations from the median within U133A and U133Plus2 array subsets). The mapping of transcripts or genes between the two array datasets was made on the Entrez Gene identifier; where multiple human array probe sets referenced the same gene, one probe set was picked at random to represent the gene (for ER gene, the probe set 205225_at was used for all Affymetrix array datasets).

For each gene-transcription profile dataset, we scored the ER^+ ^tumors for luminal A versus luminal B subtype, essentially as previously described [34], by using the dataset from Hoadley *et al*. [35] to define luminal A versus B expression patterns. In brief, for each gene common to the Hoadley platform and the other breast-array dataset platform, we computed the mean centroid of the luminal A and B subtypes in the Hoadley dataset and centered each group average on the centroid. We then took the Pearson correlation (using all genes common to both array datasets) between the Hoadley centered averages and the expression values of each profile in the independent dataset. For the ER^+ ^tumors represented on the RPPA dataset, we distinguished luminal A from luminal B tumors, by using a previously established metric (unpublished data), which relied on a panel of markers for assessing ERα function (ERα/PR/Bcl2), HER2 levels and activity (HER2/HERp1248), apoptosis (cleaved caspase 7/cleaved PARP/Bcl2), protein synthesis (p70S6K/S6 phosphorylation), cell-cycle progression (cyclin B1), and stroma (collagen VI). The expression levels of these markers from RPPA were weighted equally but in opposing directions for their association with either the luminal A (positive weighting) or luminal B (negative weighting) subtype and summed to create a classifier, by using the predefined log mean centered "luminalness" score cutoff of -0.907.

### Cell cultures

All cell lines were obtained from the American Type Culture Collection (ATCC, Manassas, VA, USA). Cell lines were cultured in RPMI 1640 (ATCC) (ZR75-1, BT483, and ZR75-B), or DMEM (Invitrogen, Carlsbad, CA, USA) (MCF7 and CAMA-1), supplemented with 10% heat-inactivated fetal bovine serum (Thermo Scientific, Pittsburgh, PA, USA) and 1% penicillin-streptomycin-glutamine (Invitrogen). Cell cultures were maintained in a humidified atmosphere of 5% CO_2 _at 37°C. For the use of PI3K inhibitor, BEZ-235 (Novartis) was added to the culture medium of a triplicate sample at a concentration of 100 n*M *or 500 n*M *at 3 hours before cell harvesting. DMSO (Sigma, St. Louis, MO, USA) with 1:1,000 dilution was used as the control. For the use of growth factor, starved cells kept in serum-free medium for 24 hours were first preincubated with DMSO (control) or BEZ-235 (100 n*M*, 500 n*M*) for 30 minutes, followed by adding 100 ng/ml of IGF-I or HCl (1:1,000 dilution) for another 3 hours before harvesting. For experiments involving estrogen deprivation, cells were cultured in phenol red-free medium supplemented with 5% charcoal-stripped fetal bovine serum (Thermo Scientific) for 48 hours before treatment.

### Quantitative real-time PCR

Total RNA was extracted with an RNeasy Mini kit (Qiagen, Hilden, Germany). One microgram RNA of each sample was reverse transcribed in a 20-μl reaction by using 200 U superscript II reverse transcriptase and random hexamers (Invitrogen). Target primer sequences are as follows: *ER-α *forward AACCGAGATGATGTAGCCAGC, reverse, CAGGAACCAGGGAAAATGTG; *PR *forward GATGCTGTATTTTGCACCTGATCTA, reverse GAACTCTTCTTGGCTAACTTGAAGCT;* CAV1 *forward GGTCAACCGCGACCCTAAA, reverse CCTTCCAAATGCCGTCAAA;* IGF1R *forward CACGACGGCGAGTGCAT, reverse ACAGACCTTCGGGCAAGGA. QPCR was performed on an ABI Prism 7500 Sequence Detection System by using SYBR Green PCR Master Mix (ABI, Foster City, CA, USA) in a 20-μl reaction and human β-actin (*ACTB*) as an endogenous control. The 20-μl reactions were incubated in a 96-well optical plate at 95°C for 10 minutes, followed by 40 cycles of 95°C for 15 seconds, and 60°C for 35 seconds. Fold changes in mRNA expression between treatments and controls were determined by the 2^-ΔΔCt ^method [36]. Differences between comparison groups were determined with a two-sided Student *t *test and one-way ANOVA. Error bars on plots represent ± standard error of the mean (SEM), unless otherwise noted.

### Growth-inhibition assays

All experiments were done in 96-well plates. Cells in quadruplicate wells were grown in regular medium and tamoxifen (Tam), BEZ-235, or Tam + BEZ-235 were added directly into each well. After 4 days, 0.05% methylene blue (Sigma) staining was performed, and the absorbance value at 650 nm was acquired by microplate reader (model 680; Bio-Rad). Growth inhibition (%) was calculated by the formula of (1 - OD_treated_/mean(OD_control_)) × 100%. Error bars for each group plotted denote the standard deviation (SD) from four independent samples. Differences between comparison groups were determined with two-sided Student *t *test and one-way ANOVA.

## Results

### A PI3K proteomic signature is associated with lower ER levels in ER^+ ^breast tumors

**Figure 1:**
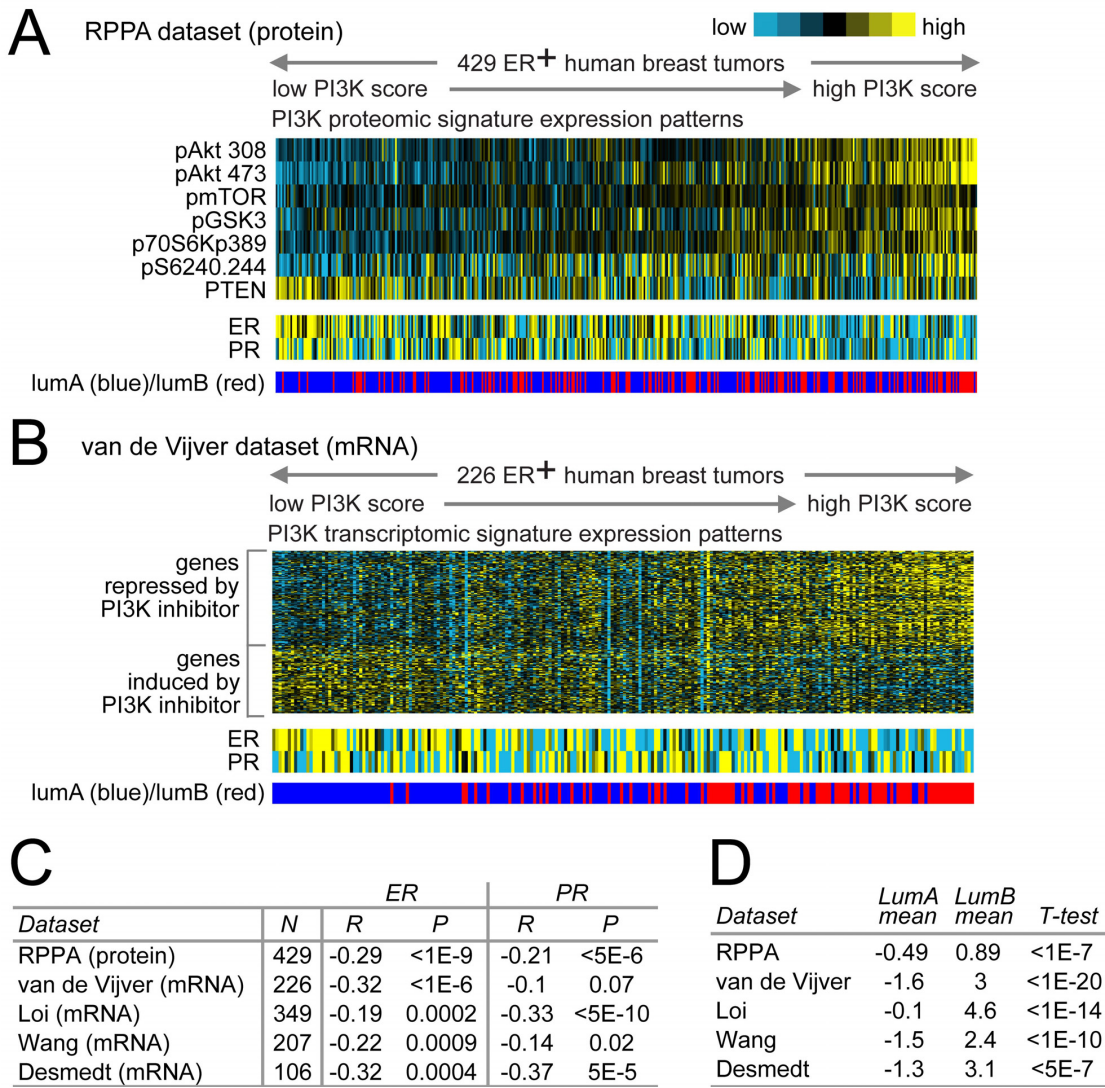
**Proteomic and transcriptomic signatures of PI3K signaling are associated in ER+ breast tumors with lower ER and PR levels and the luminal B subtype**. **(a) **Heat map of PI3K proteomic signature proteins in 429 ER^+ ^human breast tumors, along with corresponding patterns for ER and PR (blue ER on the color scale meaning lower levels, although still present) and intrinsic molecular subtype association (luminal A versus luminal B). PI3K protein score is the sum of the phosphoprotein levels of Akt, mTOR, GSK3, S6K, and S6, minus the total levels of pathway-inhibitor PTEN. Tumors are ranked from low to high PI3K score, where a high PI3K score indicates high pathway activity. **(b) **PI3K transcriptomic (that is, mRNA) signature genes in 226 ER^+ ^breast tumors (from van de Vijver *et al*.), along with ER and PR mRNA; tumors are ranked from low to high PI3K mRNA score. **(c) **Spearman's correlations between PI3K score and ER/PR in multiple expression-profiling datasets (four transcriptomic, one proteomic). For proteomic dataset, PI3K protein score and ER/PR protein levels were analyzed; for mRNA datasets, PI3K mRNA score and *ER*/*PR *mRNA. **(d) **In each of the five expression datasets, the average PI3K score in ER^+ ^tumors of the luminal B subtype was compared with the average in ER^+ ^tumors of the luminal A subtype by *t *test.

We defined a protein signature of the PI3K pathway in human ER^+ ^breast tumors by using RPPA to measure the phosphorylation states as well as total levels of key signaling intermediates of the pathway. For each of 429 ER^+ ^tumors represented on the arrays, we computed a PI3K score (Figure 1a), which was the sum of the phosphoprotein levels (mean-centered across tumors) of Akt, mTOR, GSK3, S6K, and S6, minus the total levels of pathway inhibitor PTEN (that is, PI3K score = pAkt + pmTOR + pGSK3 + pS6K + pS6-PTEN); a high PI3K score would indicate high pathway activity. Within the ER^+ ^tumors, PI3K protein signature scores were inversely correlated with ER protein levels, which pattern could be discernible by eye from heat maps of the data (Figure 1a), as well as being statistically significant (Spearman's *R *= -0.29; *P *< 1E-09). In addition to ER, ER-inducible PR was also anticorrelated with the PI3K score (Spearman's *R *= -0.21; *P *< 5E-06).

### A PI3K transcriptomic signature is associated with lower ER levels in ER^+ ^breast tumors

In addition to a proteomic signature of PI3K signaling, we defined a PI3K transcriptomic signature, representing the set of gene transcripts induced or repressed as a result of the PI3K pathway, and applied this signature to human tumors. We examined the public "Connectivity Map," or "CMap," dataset, which consists of gene-expression profiles in response to treatment by 164 different small-molecule inhibitors [37]. We compared cells treated with inhibitors for PI3K (specifically, wortmannin or LY-294002) with cells treated with other small molecule inhibitors, to define a gene-transcription signature of PI3K-inhibited cells (*P *< 0.01; FDR < 0.1, Supplemental figure S1 in Additional file 1), which consisted of 2,221 Affymetrix probe sets (755 unique genes induced by the inhibitors, 1,046 genes repressed; complete gene list in Additional file 2). In addition to the CMap PI3K signature, we also considered two other gene signatures, one of PTEN loss in human breast tumors [38] and another of Akt overexpression in mouse [13,39]. We found that these three signatures were highly correlated with each other in terms of the same breast-tumor samples showing high PI3K activity (Supplemental Table 1 in Additional file 1), although all subsequent results shown here make use of the CMap signature.

We applied the CMap PI3K mRNA signature to a public gene-expression profile dataset of 226 human ER^+ ^breast tumors from van de Vijver *et al*. [40], scoring each tumor for PI3K signature manifestation (Figure 1b). As the CMap patterns were of PI3K inhibition, those tumors positively correlated with these patterns were inferred to have low PI3K activity, and those tumors anticorrelated with these patterns were inferred to have high PI3K activity. Within the van de Vijver ER^+ ^tumors, the PI3K mRNA signature scores (a high score indicating high pathway activity) were inversely correlated with ER mRNA levels. These patterns could be discernible by eye (Figure 1b) as well as being statistically significant (Spearman *R *= -0.32, *P *< 1E-06). In addition to the van de Vijver dataset, we examined three other independent gene-expression datasets of ER^+ ^tumor from other studies [41-43], in which a pattern of inverse correlation between PI3K score and ER mRNA was statistically significant there as well (Figure 1c). PR mRNA was also significantly anticorrelated with the PI3K score in three of the four mRNA datasets (*P *< 0.05) and was trending toward significance in the fourth dataset (*P *= 0.07) (Figure 1c). In summary, the association of high PI3K activity with lower ER and PR appeared to be quite robust, and the results of the PI3K mRNA signature agreed with those of the PI3K protein signature.

### PI3K proteomic and transcriptomic signatures are associated with the luminal B molecular subtype of ER+

Gene-expression profiling of human breast tumors has been used to classify them into several distinct and clinically relevant groups (basal-like, luminal A, luminal B, HER2^+^/ER^-^, and normal-like) [44]. In particular, ER^+ ^tumors can be subdivided into the less-aggressive luminal A subtype and the more-aggressive luminal B subtype. In each of the expression datasets examined (four transcriptomic, one proteomic), we scored the ER^+ ^tumors for luminal B versus luminal A subtype, and we found that the PI3K signature scores (both protein and mRNA) were significantly higher in luminal B tumors (Figure 1d). Furthermore, in the two transcriptomic datasets for which patient-outcome and patient-treatment information were available (van de Vijver and Loi), the PI3K mRNA signature predicted worse prognosis in ER^+ ^tumors (Supplemental figure S2 in Additional file 1); this trend of worse prognosis for tumors with high PI3K score also was evident in the subset of patients that received hormone therapy as well as in untreated patients.

### PI3K proteomic and transcriptomic signatures are correlated within breast cancer cell lines

**Figure 2:**
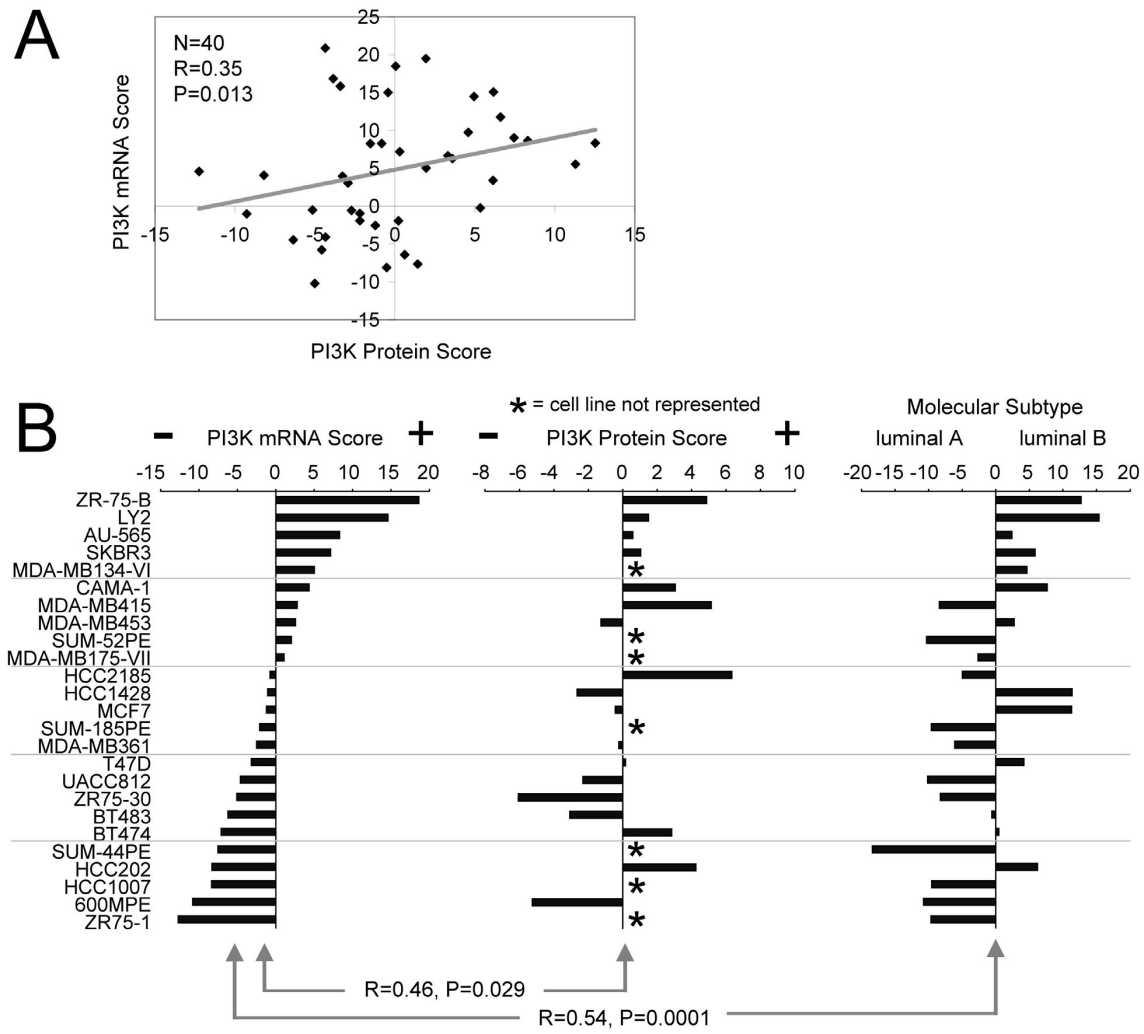
**PI3K protein and mRNA activation scores correlate with each other and with the luminal B subtype in breast cancer cell lines**. **(a) **Scatterplot of PI3K protein score versus PI3K mRNA score in 40 breast cancer cell lines. **(b) **Luminal breast cancer cell lines (as defined by Neve *et al*.) plotted alongside their PI3K mRNA score (left) and their PI3K protein score (middle), along with their correlation with a luminal B signature from Hoadley *et al. *(right). Note that some of the cell lines were not analyzed by proteomics and are indicated (*). Correlations (*R *values) by Spearman's.

We went on to examine the PI3K signatures in breast cancer cell lines, where we had both gene-expression data (from Neve *et al*. [45]) and proteomic data (from our group) on the same set of 40 cell lines. Even given this limited number, the PI3K protein score correlated significantly (albeit not perfectly) with the PI3K mRNA score across all cell lines (Spearman's *R *= 0.35; *P *= 0.013; Figure 2a), as well as within the subset of cell lines previously defined as "luminal" (that is, associated with ER^+^) in subtype (n = 18; *R *= 0.46; *P *= 0.029; Figure 2b). In addition, we scored the 25 luminal cell lines in the Neve mRNA dataset for similarity to the luminal B versus luminal A subtype patterns, and, as observed in the tumors, the PI3K mRNA scores in luminal cell lines tended to associate with luminal B (*R *= 0.57; *P *= 0.001; Figure 2b). Of the luminal cell lines examined here, 12 were recently examined in a previous study, for mutations in *PTEN *and *PIK3CA *[24]; however, as 11 of the 12 cell lines were found to harbor mutations in either one of the two genes, no correlations were apparent between PI3K pathway alteration by mutation and luminal B or PI3K signature scores.

### Modulation of PI3K signaling in breast cancer cell lines has an inverse effect on levels of ER and ER-inducible genes

**Figure 3:**
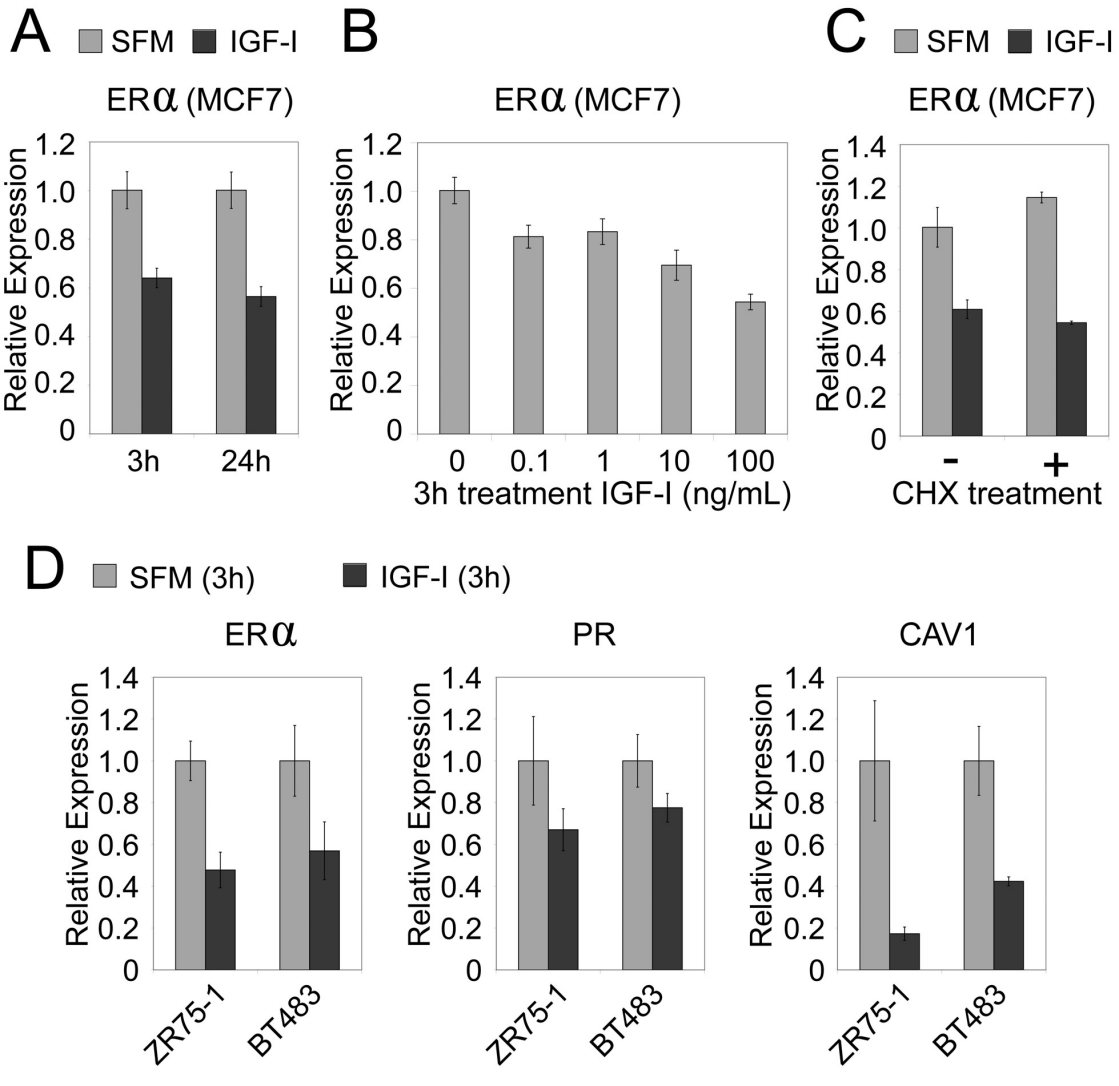
**PI3K-activator IGF-I rapidly downregulates mRNA levels of ER and ER target genes. (a) **MCF-7 breast cancer cells were starved in SFM and then incubated with IGF-I (100 ng/ml, 8 n*M*) for 3 or 24 hours. RNA was isolated, and ER mRNA levels were measured with QRT-PCR (± SEM). **(b) **MCF-7 cells were incubated with an increasing dose of IGF-I, and ER mRNA levels were measured as in part **(a)**. **(c) **ER levels in MCF-7 cells incubated with (+) or without (-) cycloheximide and stimulated with or without IGF-I (100 ng/ml) for 3 hours. **(d) **In two additional cell lines (ZR75-1 and BT483), expression levels of ER and ER target genes (*PR *and *CAV1*) after IGF-I treatment (100 ng/ml for 3 hours). All comparisons shown here between IGF-I (100 ng/ml) and control (± SD) were significant, with *P *≤ 0.05.

Although our analysis of molecular signature patterns of PI3K in human tumors showed at least a correlative (inverse) relation between PI3K and ER, we could also demonstrate a *functional *relation between the two, by using cell-culture models. With gene-profiling data from cell lines (Figure 2), we selected a number of cell lines for further functional studies, including ZR75-B and CAMA-1, which scored highly for both PI3K and luminal B expression patterns, and ZR75-1 and BT483, which had low PI3K scores and associated more with luminal A. In many systems, IGF-I (through its receptor IGF-IR) is a potent activator of PI3K [46]. We previously showed in MCF-7 cells that IGF-I activates PI3K/Akt/mTOR to downregulate PR mRNA levels rapidly through direct inhibition of PR promoter [46]. Similarly, we report here that treatment of MCF-7 cells with IGF-I caused a dose-dependent rapid reduction in ER mRNA levels within 3 hours, and this reduction remained constant over a 24-hour period (Figure 3a). This downregulation was dose responsive (Figure 3b), and the rapid reduction was a direct effect of IGF-I, as it was not affected by incubation of the cells with cycloheximide (Figure 3c) and thus does not require new protein translation. We also found in two additional cell lines tested (ZR75-1 and BT483) that IGF-I decreased expression of *ER *and ER target genes *PR *and *CAV1 *(Figure 3d).

**Figure 4:**
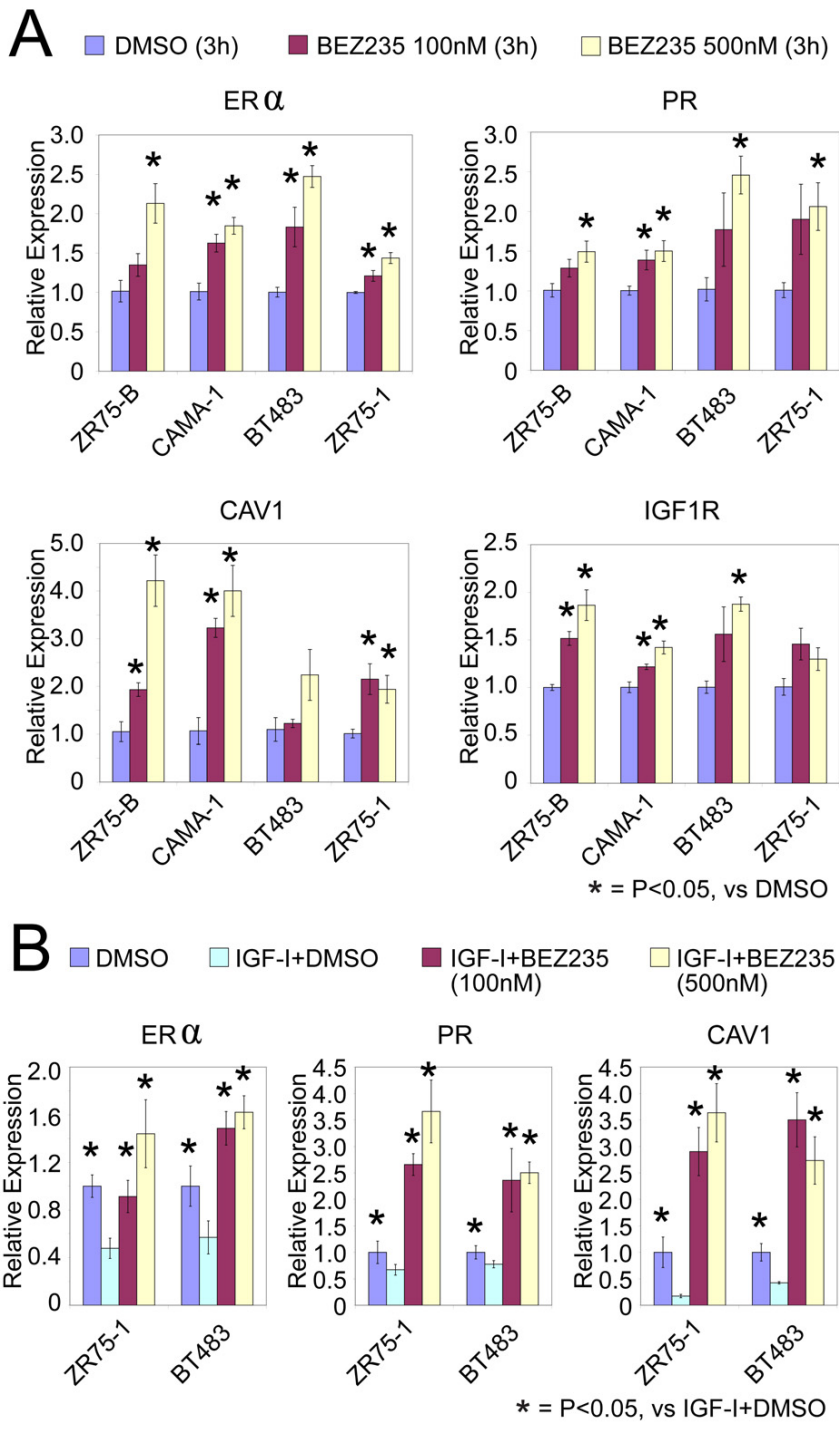
**Inhibition of PI3K signaling increases mRNA levels of ER and ER-inducible genes. (a) **QRT-PCR results showing increased expression of *ER *and ER target genes (*PR*, *CAV1*, and *IGF1R*) in four different cell lines, after treatment with PI3K inhibitor BEZ-235 (100 n*M *and 500 n*M *for 3 hours). *Significant differences (*P *< 0.05; *t *test) between BEZ-235 and control (multiple comparisons expected to yield 32 × 0.05/23 = 7% false positives). Twelve of the 16 marker/cell-line combinations individually significant (*P *< 0.05) by ANOVA (*P *> 0.05: *PR*/ZR75-B, *PR*/ZR75-1, *CAV1*/BT483, *IGF1R*/ZR75-1; false-positive rate: 16 × 0.05/12 = 7%). **(b) **IGF-I-mediated downregulation of ER and ER target genes is restored by inhibition of PI3K. ZR75-1 and BT483 cells were preincubated for 30 minutes with or without BEZ-235 (100 and 500 n*M*) and then stimulated with or without IGF-I (100 ng/ml) for 3 hours. ER and ER targets gene mRNA levels were measured with QRT-PCR (data from DMSO and IGF-I + DMSO groups also are featured in Figure 3a). *Significant differences from corresponding IGF-I + DMSO group (*P *≤ 0.05; t test). All marker/cell-line combinations were significant (*P *< 0.03) by ANOVA.

Consistent with the preceding section, inhibiting PI3K had the opposite effect on the ER from stimulating the PI3K pathway by IGF-I. In four different ER^+ ^breast cancer cell lines tested (two of them luminal A and two of them luminal B), treatment with PI3K inhibitor BEZ-235 for 3 hours significantly increased expression of *ER *and ER-inducible target genes (*PR*, *CAV1*, and *IGF1R*); this result was observed by using two concentrations of the inhibitor (100 n*M *and 500 n*M*), with the higher concentration appearing to have a slightly more dramatic effect on the genes (Figure 4a). As expected, BEZ-235 downregulated phosphorylation of key PI3K signaling intermediates included in our PI3K protein signature (Supplemental figure S3 in Additional file 1). Interestingly, in addition, BEZ-235 had a suppressive effect on estrogen-driven cell growth (Supplemental figure S4 in Additional file 1), indicating that ER signaling is also at least somewhat dependent on PI3K; this can be partly explained by the fact that plasma-membrane-associated ER (acting nongenomically) is able to activate various GFRs and PI3K [47].

**Figure 5:**
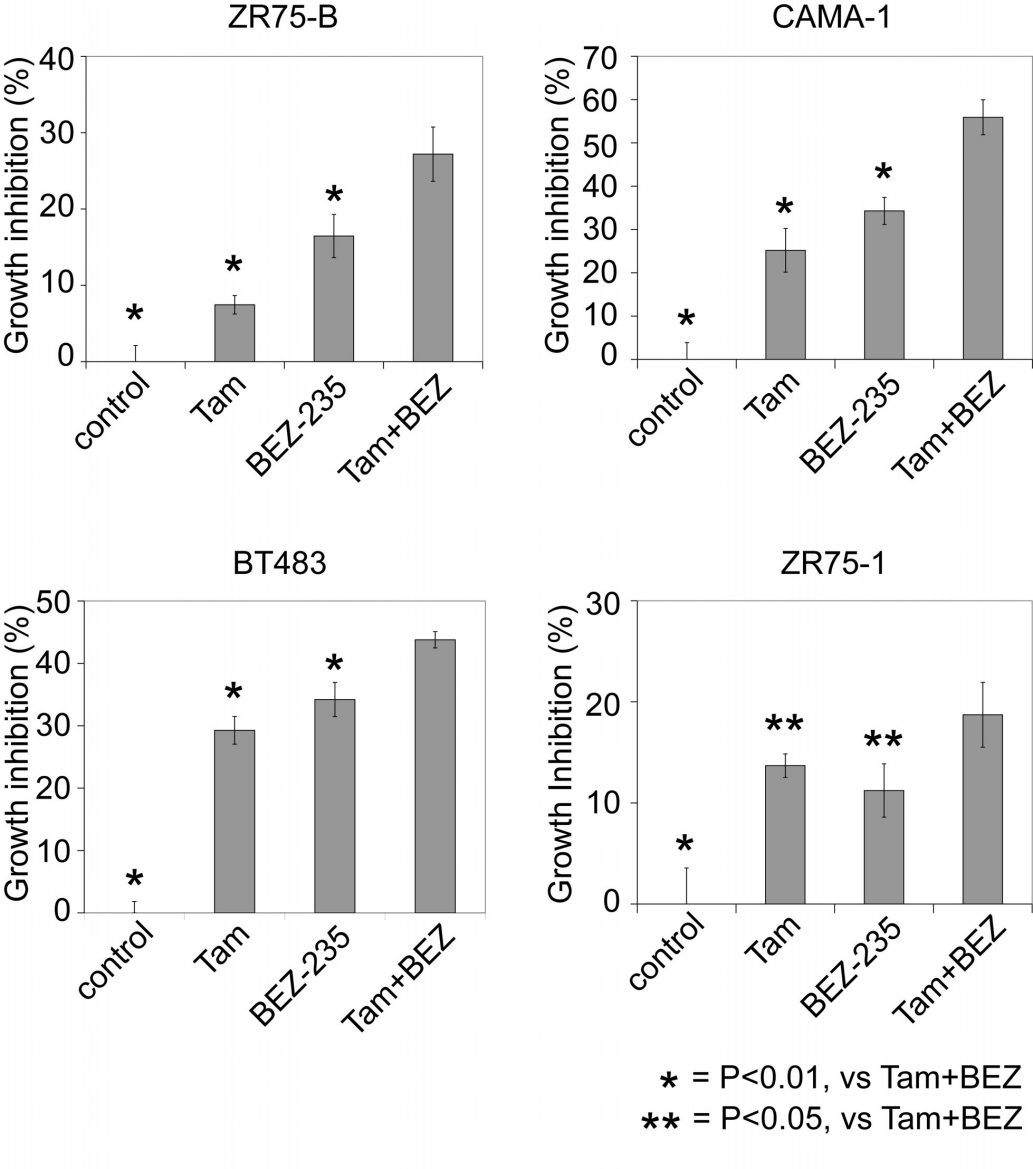
**PI3K inhibitor BEZ-235 has an additional effect on tamoxifen (Tam) in ER^+ ^cell lines**. Growth-inhibition assays for luminal B-cell lines ZR75-B and CAMA-1 and luminal A cell lines BT483 and ZR75-1, treated for 4 days with Tam (100 n*M*), BEZ-235 (10 n*M*), or the combination of Tam + BEZ-235. Average growth inhibition normalized to control (regular medium) in each cell line (± SD). *Significant differences from Tam + BEZ-235 group (*P *< 0.05; *t *test). For each cell line, significant differences exist among the groups by ANOVA (*P *< 1E-5).

Consistent with IGF-I-reducing levels of ER through the PI3K pathway, treatment of IGF-I-stimulated cells with BEZ-235 could increase levels of *ER *and ER target genes relative to their IGF-I-repressed levels (Figure 4b), showing that PI3K is required for IGF-I-mediated downregulation of ER expression and activity on classic ER-dependent gene transcription. Similar results were found with MCF-7 cells (data not shown). Increasing ER levels and activity by inhibiting PI3K should presumably increase hormone sensitivity, and in four different cell lines tested (two of them luminal A, and two of them luminal B), the combination of BEZ-235 and tamoxifen inhibited growth more than either tamoxifen alone or BEZ-235 alone (Figure 5).

## Discussion

In this study, we found that GFR/PI3K signaling is associated in ER^+ ^breast cancers with relatively lower ER levels and with the luminal B molecular subtype. It is worthy of note that the lower ER levels in those ER^+ ^tumors with high PI3K activity were still detectable, as these tumors were still clinically defined as ER^+^. Evidence for the link between PI3K and ER was found here both by using molecular "signatures" of PI3K to probe human ER^+ ^tumors and by manipulating the PI3K pathway in cell-culture models. Importantly, ER levels and activity could be increased in cell cultures by blocking the PI3K pathway. Our interpretation of these data is that some ER^+ ^tumors rely more heavily on GFR/PI3K signaling than on estrogen for growth, and that by blocking PI3K, these tumors would be forced to resort to the alternative estrogen-signaling pathway for continued growth; by blocking both PI3K and estrogen pathways together, therefore, the tumor may be left with even fewer options.

As the luminal B subtype is the much more aggressive subtype of ER^+ ^breast cancer [44], targeting PI3K in these tumors might reverse loss of ER expression and signaling and restore hormonal sensitivity. In addition to luminal B cancers, many basal-like (ER^-^/PR^-^/HER2^-^) cancers have loss or mutation of *PTEN *and high PI3K activity [11,33], and some (though not all) basal cancers can reactivate the ER in response to GFR inhibition [48]. The two ER^- ^cell lines we have examined to date (MDA-MB-231 and MDA-MB-468) did not reexpress ER in response to BEZ-235 (data not shown), although this could be another avenue for future work.

In selecting luminal/ER^+ ^cell lines for study, one could conceivably use our scoring for PI3K signature activation as a guide, although it remains to be seen how cell lines with high PI3K scores might behave differently from cell lines with low scores. One could hypothesize that PI3K scores are an indicator of response to therapies targeting the PI3K pathway; however, to date, we have not found evidence of this in our 2-D culture models. In one recent study by Brachmann *et al*. [49], a panel of breast tumor cell lines was treated with BEZ-235; however, as the observed 50% growth inhibition (GI-50) values were all in the low nanomolar range, the authors concluded that BEZ-235-induced growth inhibition in the 2-D setting was not amenable for stratification prediction. Consistent with this notion, we obtained the GI-50 values from Brachmann *et al*., but could not find any trend for correlation with our PI3K scores (neither protein nor mRNA). Furthermore, we made a point of manipulating the PI3K pathway in both cell lines with high PI3K scores and cell lines with low scores, and both sets of cell lines appear to yield similar results. It is important to keep in mind that our PI3K scores represent a *relative *rather than an absolute measure of PI3K activity, and it appears that the functional relation between PI3K and ER exists to at least some degree in most ER^+ ^cancers. Nevertheless, the PI3K scoring might prove relevant in model systems beyond 2-D or in measures other than growth.

The dynamic nature of ER and PR levels in human breast cancer, and the potential to alter levels for therapeutic benefit, has recently gained much interest [48]. Levels of ER are known to correlate significantly correlate with patients' response to endocrine therapy, and quantitative ER measurement with RT-PCR has been shown to be the best single predictor of tamoxifen benefit [50]. Reduced expression or a complete loss of ER may occur at multiple levels and by multiple mechanisms, from the gene to the protein [51]. ER levels are controlled in a homeostatic fashion by many interacting pathways. For example, ER mRNA and protein can be downregulated in MCF-7 cells by stably overexpressing EGFR or constitutively activating erbB-2, Raf, or MEK [52]; and in a number of ER^- ^breast cancers, ER expression can be restored by inhibiting GFR through targeting of MAPK/ERK [48]. Of interest to this study, the Forkhead box class O (FoxO) family members, which are known downstream targets of PI3K, have recently been shown to play a major role in modulating both ER levels and activity. For example, FOXO3A (in our PI3K mRNA signature, one of the genes showing repression by PI3K (i.e., induction by PI3K inhibitors) can bind the ER promoter and increase ER levels, and HER-2/Akt-mediated activation and translocation of FOXO3A out of the nucleus results in a reduction of ER levels [53]. Interestingly, we found *FOXO3A *to be underexpressed (based on gene array) in MCF-7 xenograft tumors that had developed resistance to tamoxifen [54]. Besides FOXO3A, other master transcription factors, including Snail, can modulate ER promoter activity [55], and we have shown that IGF-IR through PI3K can elevate Snail [56].

## Conclusions

Our study implies that ER^+ ^patients with high GFR/PI3K signaling, who presumably are at greater risk of developing resistance to hormone therapy alone, may need to be treated with GFR/PI3K-targeted therapy in addition to hormone therapy. Clinical trials are currently under way for PI3K inhibitors such as BEZ-235. As was found to be the case with therapies targeting HER2 or ER, defining the patient population most likely to respond to PI3K-targeting therapy may well prove critical in establishing the success of these new drugs. In this regard, clinical studies of PI3K inhibitors that focus on the subset of ER^+ ^patients with either tumors of the luminal B molecular subtype (which could be defined, for example, by using the PAM50 clinical assay [57]) or a prediction of poor outcome on hormone therapy alone (for example, as defined by using the Genomic Health Index [58]), could provide valuable information on targeting the PI3K pathway in breast cancer.

## Abbreviations

BC: breast cancer; ER: estrogen receptor (alpha); GFR: growth factor receptor; IGFR: insulin-like growth-factor receptor; PI3K: phosphoinositide 3-kinase; PR: progesterone receptor; RPPA: Reversed Phase Proteomic Array.

## Competing interests

AMG has an investigator initiative study sponsored in part by Novartis. The other authors have no competing interests to disclose.

## Authors' contributions

CJC, AVL, and RS contributed to concept design, data analysis and interpretation, and manuscript writing. XF contributed to concept design, generation of experimental data, data analysis and interpretation, and manuscript writing. BTH and GBM contributed to concept design, generation of proteomic data, and data analysis and interpretation. AJC contributed to generation of experimental data and data analysis. SGH contributed to concept design and data analysis and interpretation. AMG contributed to generation of proteomic data. YZ contributed to data analysis. CKO contributed to concept design and data interpretation. AL and PHB contributed human tumor specimens. JWG contributed protein lysates from cell lines. All authors read and approved of the final manuscript.

## Supplementary Material

Additional file 1 **Supplemental Figures 1 through 4 and Supplemental Table 1**. Click here for file

Additional file 2 **Genes in the transcriptomic signature of PI3K signaling**. Complete set of genes in the transcriptomic signature of PI3K signaling, based on the Connectivity Map (CMap) dataset. Click here for file
