# Supplementary material for: Proteomic and transcriptomic profiling reveals a link between the PI3K pathway and lower estrogen-receptor (ER) levels and activity in ER+ breast cancer
Source: Breast Cancer Res. 2010 Jun 22;12(3):R40. doi: 10.1186/bcr2594 (PMC2917035; doi:10.1186/bcr2594)
Supplement: Additional file 1 — Supplemental Figures 1 through 4 and Supplemental Table 1. [file bcr2594-S1.PDF]

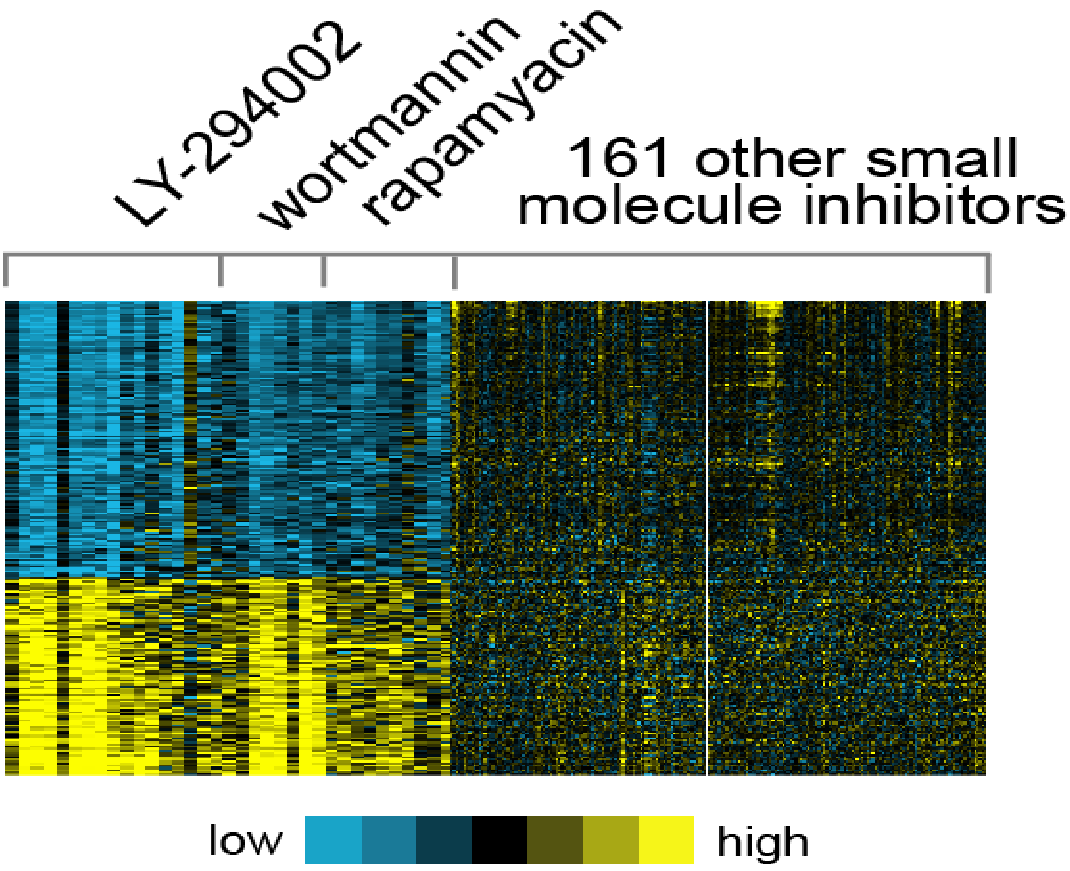

Supplemental Figure 1. Transcriptomic signature of PI3K signaling, based on the “Connectivity Map” (CMap) dataset. Using a compendium of expression profiles from cell lines treated with 164 different small molecule inhibitors, profiles of cells treated with inhibitors for PI-3K were compared with profiles of cells treated with some other small molecule inhibitor (selected using nominal $P<0.01$, 2221 Affymetrix probe sets selected, yielding $FDR<0.1$). Profiles for PI3K inhibitors LY-294002 and wortmannin and the mTOR inhibitor rapamyacin are highlighted (the rapamyacin profiles themselves not being used in defining the signature). Yellow: high expression; blue, low expression.

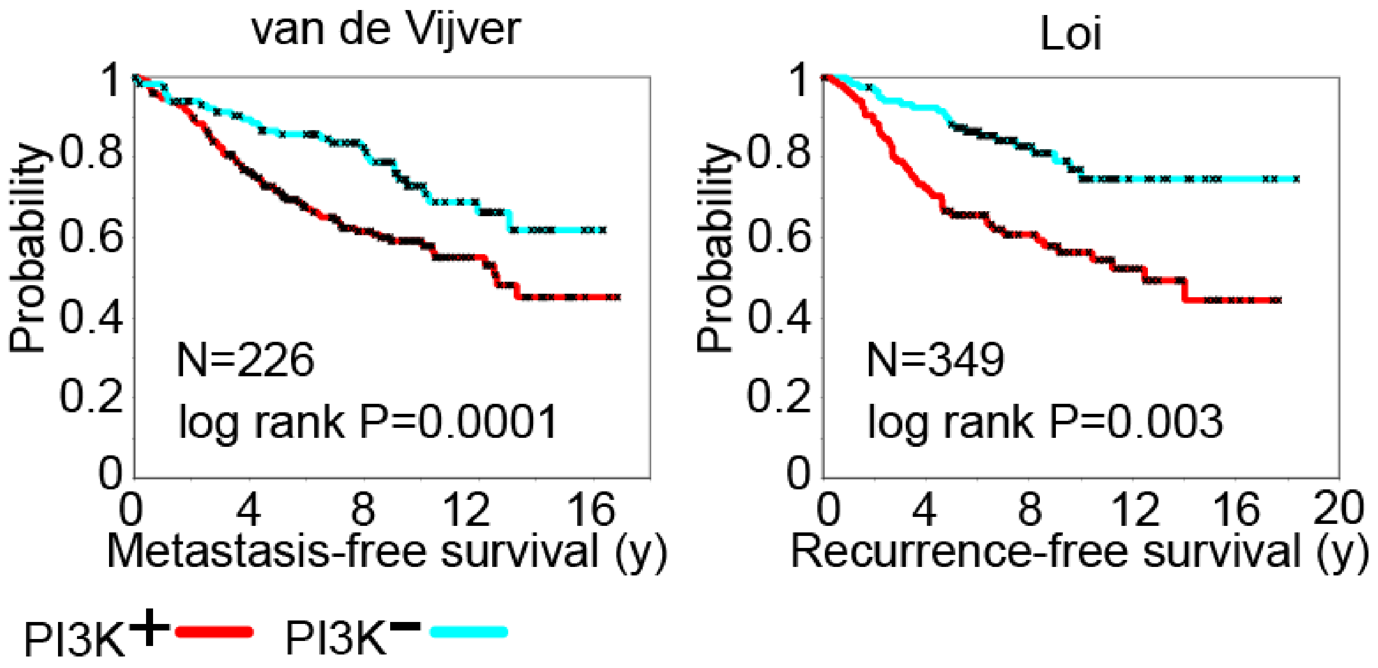

Supplemental Figure 2. PI3K mRNA signature predicts worse prognosis in ER+ tumors. Kaplan-Meier analysis (using log-rank tests) comparing the differences in risk of poor outcome event between ER+ tumors showing high PI3K activation (red line, PI3K mRNA score>0) and ER+ tumors showing low PI3K activation (blue line, PI3K mRNA score<0). Two gene expression datasets are represented here, one from van de Vijver *et al*. (left panel) and one from Loi *et al*. (right panel). All patients with ER+ tumors from each of these datasets, regardless of adjuvant treatment, are represented here.

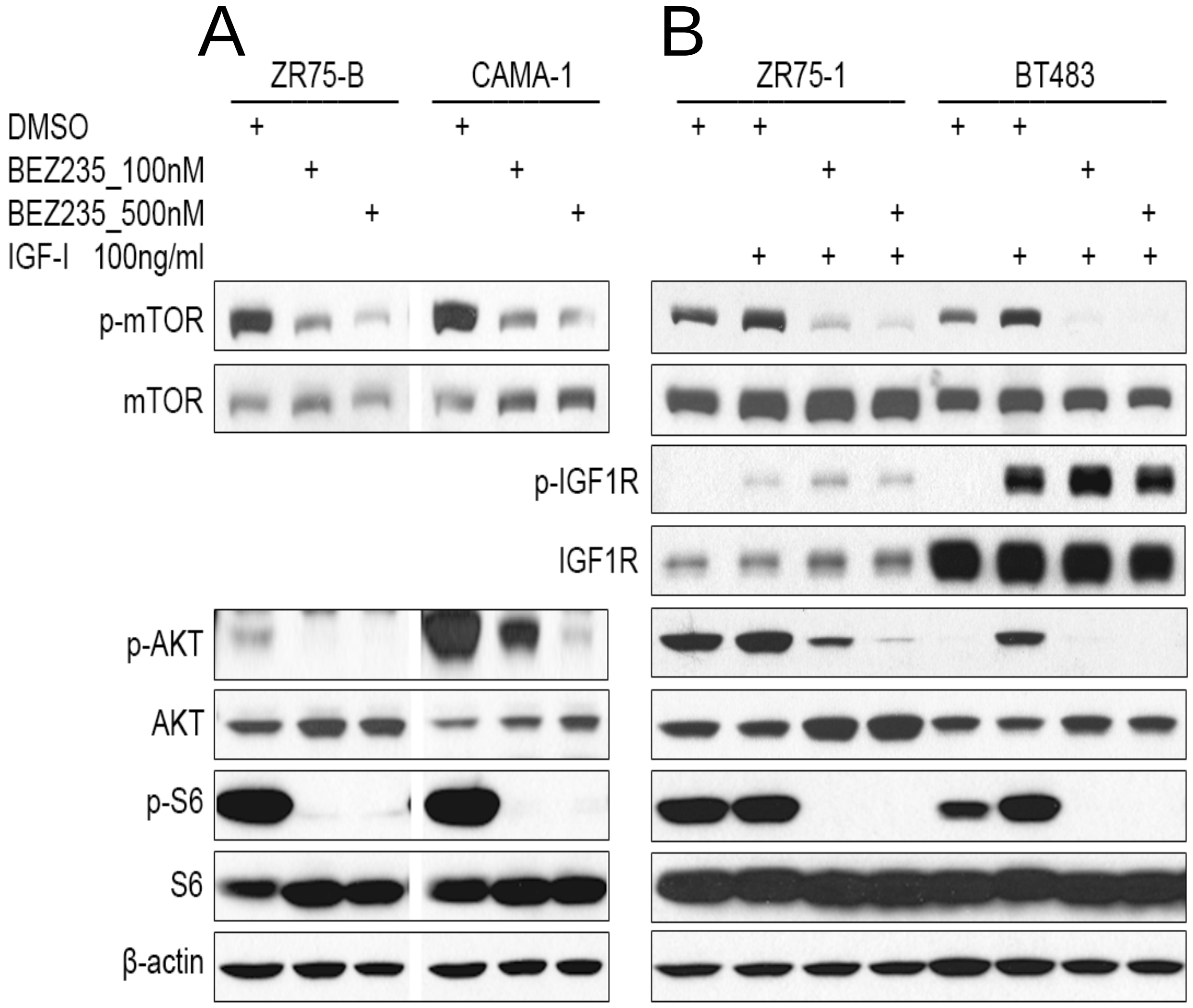

Supplemental Figure 3. PI3K inhibitor BEZ-235 downregulates phosphorylation of key PI3K signaling intermediates included in our PI3K protein signature. (A) ZR75-B and CAMA-1 (luminal B, high PI3K scoring) cells were treated with or without BEZ-235 (100nM and 500 nM). (B) ZR75-1 and BT483 (luminal A, low PI3K scoring) cells were preincubated for 30 mins with or without BEZ-235 (100nM and 500 nM) and then stimulated with or without IGF-I (100ng/mL) for 3hrs. Shown here are immunoblots of levels of both total and phosphorylation levels of mTOR, Akt, and S6. In addition, as expected, treatment with IGF-I increased phosphorylation of IGF-1R.

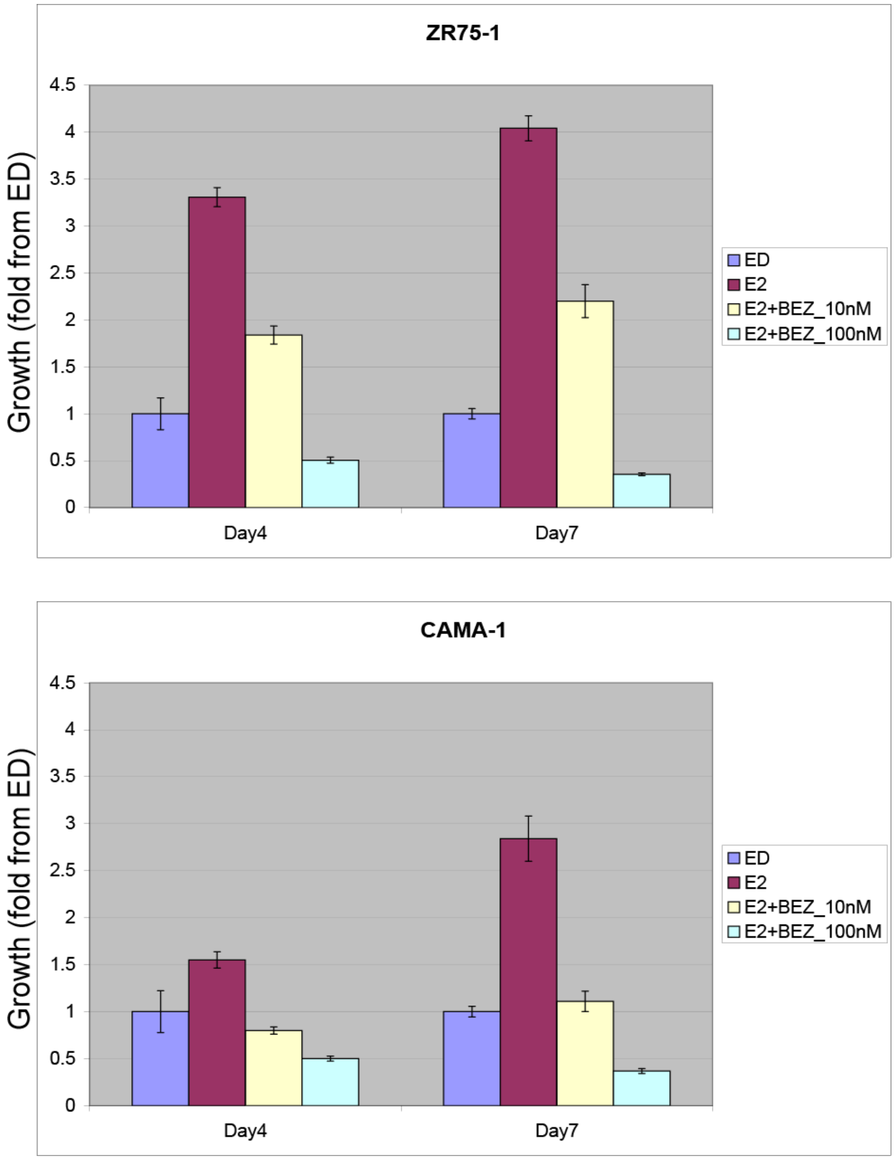

Supplemental Figure 4. BEZ-235 has a suppressive effect on estrogen-driven cell growth. Growth-curve assays for ZR75-1 and CAMA-1 under conditions of estrogen deprivation (ED, i.e. pre-starvation in estrogen-deprivation, low-growth factors medium) and treatment by E2 (1nM), with or without BEZ-235 (10nM and 100nM). These experiments were done in 96-well plate. Cells were kept in 5% CS-FBS + PRF medium for 48 hr, before treatment. At day 4 and 7, methylene blue staining was performed and raw numbers were normalized to ED treatment in each group. Error bars represent SD of triplicate samples.

Supplemental Table 1. Correlations between mRNA scores from different PI3K signatures within the 226 van de Vijver ER+ tumors.

			Correlation (R-value)		
**Author**	**Model**	**Genes**	**Lamb**	**Saal**	**Majumder**
Lamb	CMap, PI3K inhibitor	1801	1	0.71	0.75
Saal	PTEN loss, breast tumors	173	0.71	1	0.7
Majumder	Akt+ mouse prostate	770	0.75	0.7	1

(Saal signature, from Saal et al. PNAS, 2007, 104:7564-9; Majumder signature, P<0.001 WT vs Akt-overexpression. P<1E-33 each Spearman's R-value.)
